# Supplementary material for: The Grueneberg ganglion controls odor-driven food choices in mice under threat
Source: Commun Biol. 2020 Sep 24;3:533. doi: 10.1038/s42003-020-01257-w (PMC7518244; doi:10.1038/s42003-020-01257-w)
Supplement: Supplementary file 1 — Supplementary Information [file 42003_2020_1257_MOESM1_ESM.pdf]

Supplementary Information for

The Grueneberg ganglion controls odor-driven food choices in mice under threat

Julien Brechbühl, Aurélie de Vallière, Dean Wood, Monique Nenniger Tosato and Marie-Christine Broillet.

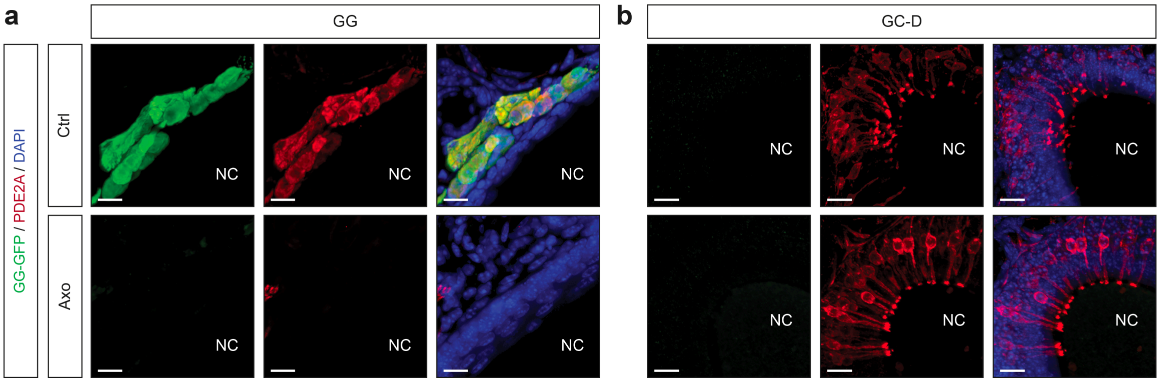

Supplementary Fig. 1 GCG–Cre–GFP mice highlight intertwined GG and GC-D circuitries. **a-b** Representative immunostainings obtained from Ctrl and Axo GCG–Cre–GFP mice (anti-GFP, GG-GFP, in green; anti-PDE2A, PDE2A, in red). GG axotomy leads to the deletion of GG neurons (a) and their afferent glomeruli without affecting the GC-D neurons (b) and their affiliated glomeruli. Nasal cavity: NC. Scale bars are, 10 μm (a) and 20 μm (b). DAPI counterstains are shown in blue.

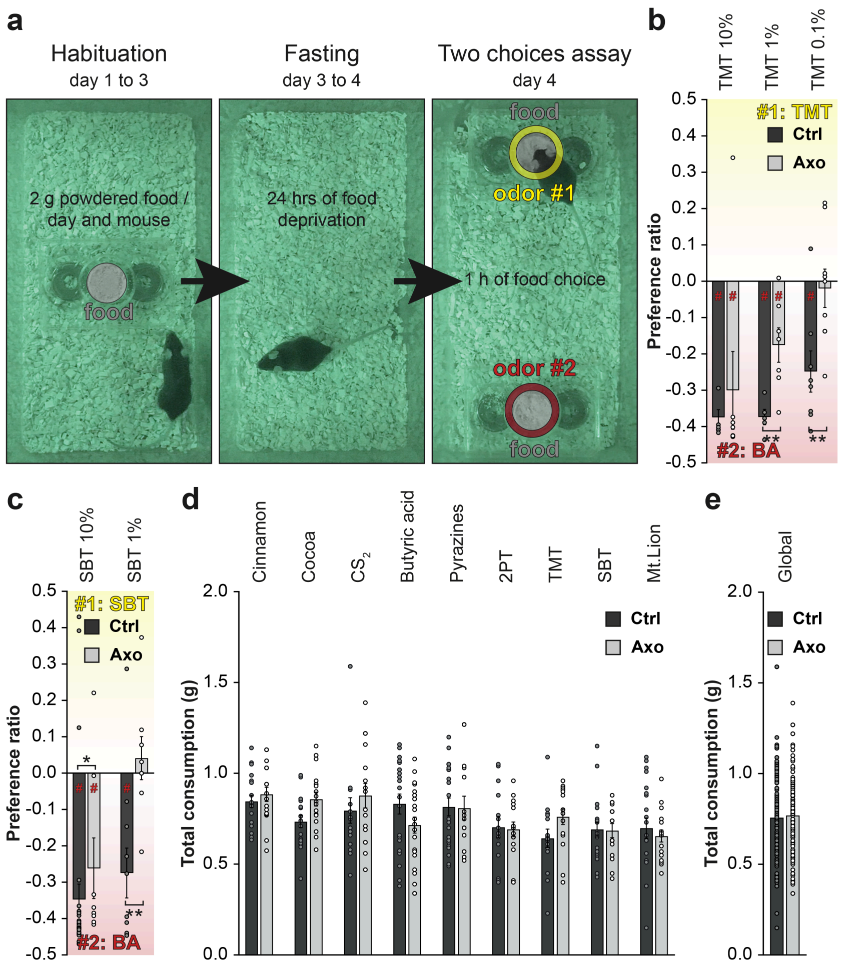

Supplementary Fig. 2 Two choices assay detailed procedure. **a** Extended illustration of the two choices assay with infrared snapshots. Details of the habituation and of the time procedure are indicated. Soiled odorants were placed around the food (odor #1, yellow; odor #2, red). **b-c** Details of the quantifications of food preference ratio for Ctrl and Axo mice obtained with diluted doses of the fox scent 2,4,5-trimethylthiazoline ((b); TMT, 10%, 1% and 0.1%) and the mouse alarm pheromone 2-*sec*-butyl-4,5-dihydrothiazole ((c); SBT, 10% and 1%) as odor #1 versus the aversive BA (10%) as odor #2. Calculation of statistical significances of preference ratio is performed with Z tests, # $p < 0.05$; non-significant if not mentioned. **d-e** The total food consumption for indicated tested cue (d) or globally (e) is illustrated. (b-e) Values obtained from Ctrl (black) and Axo (grey) mice are represented as mean ± SEM with aligned dot plots. For comparisons between Ctrl *vs.* Axo mice, two-tailed Student's t-tests or Wilcoxon w-tests are used, * $p < 0.05$; ** $p < 0.01$.

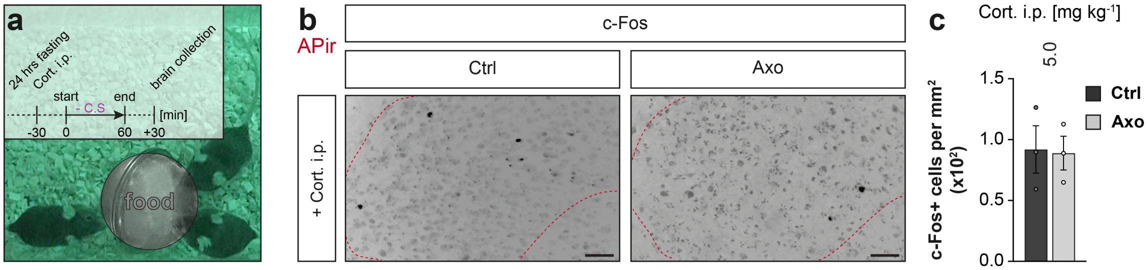

Supplementary Fig. 3 Intraperitoneal injection of corticosterone does not activate the APir area. **a** Infrared snapshot and procedure time-table illustrating mice in contact with an unreachable food resource (food, grey) without conditioning stimuli (- C.S). **b** Representative c-Fos activity observed in the APir area (dashed red lines) after intraperitoneal injection of 5.0 mg kg^{-1} of corticosterone (+ Cort. i.p.). **c** Quantification of the density of c-Fos+ cells observed in APir. Values obtained from Ctrl (black) and Axo (grey) mice are represented as mean ± SEM with aligned dot plots. 3 animals were used per Ctrl and Axo mice.

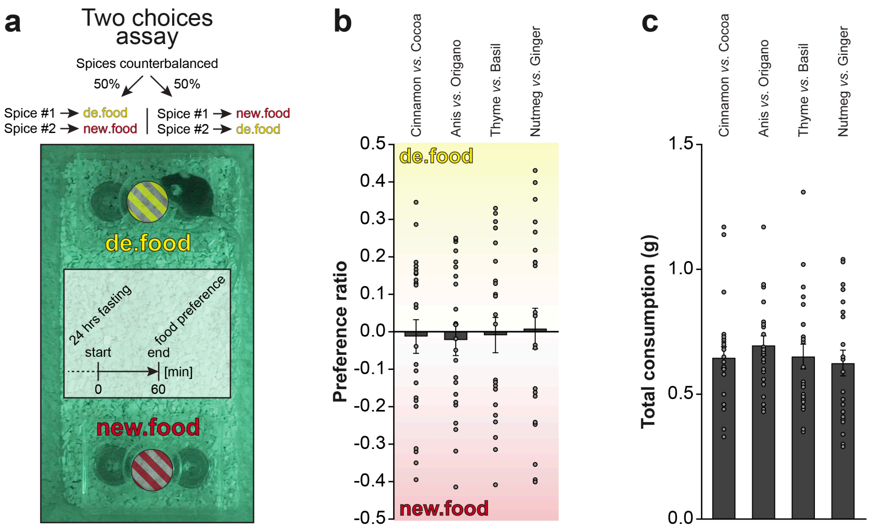

Supplementary Fig. 4 Selection of spices without innate preferences when used in a two choices assays performed under counterbalanced mode. **a** Schematic representation with an infrared snapshot and a procedure time-table of the two choices assay performed to evaluate each pairs of spices (Spice #1 *vs*. Spice #2) delivered within the powdered food. The demonstrated spice (de.food, yellow) and the novel one (new.food, red) are counterbalanced within pairs. **b** Quantification of food preference ratio for mice with the following pairs of spices in counterbalanced mode: Cinnamon (1%) *vs*. Cocoa (2%); Anise (1%) *vs*. Oregano (2.4%); Thyme (2%) *vs*. Basil (1.4%); Nutmeg (1%) *vs*. Ginger (1%). Calculation of statistical significances of the preference ratio performed with Z tests lead to non-significant preferences. **c** The total food consumption for indicated tested pairs of spices in counterbalanced mode. (b-c) Data are represented as mean ± SEM with aligned dot plots. A minimum of 22 mice are used per condition.

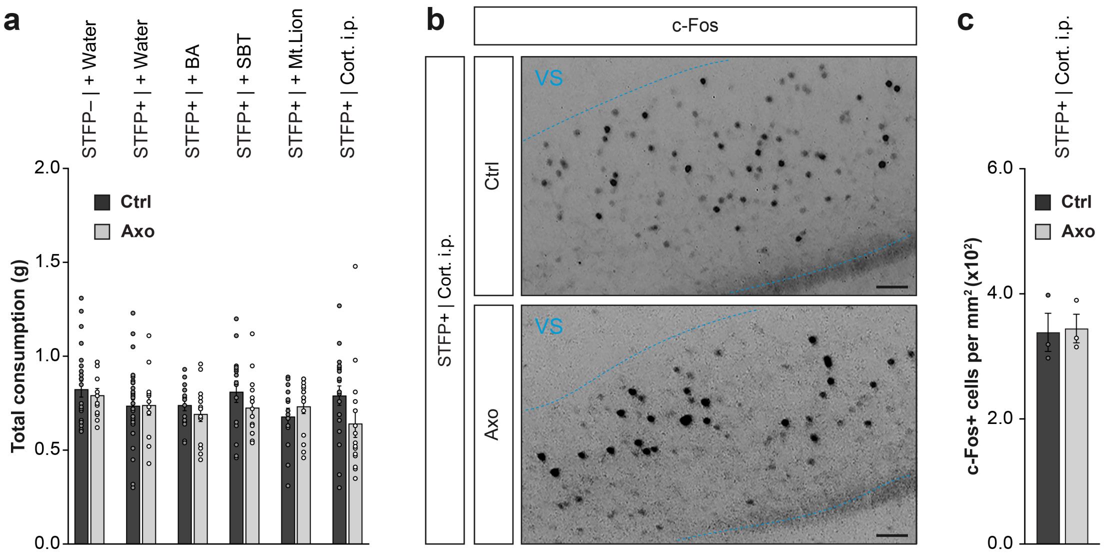

Supplementary Fig. 5 Enhancement of an acquired food preference by chemical danger cues is not induced by a modification of the total food consumption or by a cortico-dependent VS activity. **a** The total food consumption of mice without or with STFP procedure and under the indicated environmental conditioning (STFP– or + | + C.S) or under intraperitoneal injection of 5.0 mg kg^{-1} corticosterone (Cort. i.p.) are shown. 12 to 36 animals were used per condition. The following pairs of spices were used: cinnamon 1% *vs.* cocoa 2%; anise 1% *vs.* oregano 2.4%; thyme 2% *vs.* basil 1.4%. **b** Representative c-Fos activity observed in the VS area (dashed blue lines) under STFP+ | Cort. i.p. condition. **c** Quantification of the density of c-Fos+ cells observed in VS under STFP+ | Cort. i.p. condition. 3 animals were used per Ctrl and Axo mice. (a and c) Values obtained from Ctrl (black) and Axo (grey) mice are represented as mean ± SEM with aligned dot plots.

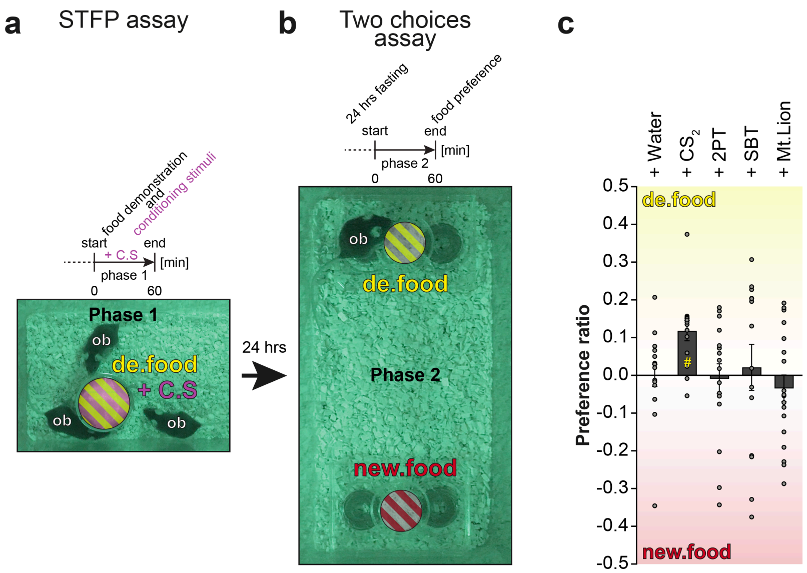

Supplementary Fig. 6 Absence of direct transmission of food preference/avoidance by GG-related cues. **a-b** Schematic representation with infrared snapshots and procedure time-tables of the STFP assay (a, Phase 1) followed by the two choices assay (b, Phase 2) performed to evaluate food preference of observer mice (*ob*). The demonstrating food (de.food, in yellow) is in the Phase 1 directly moistened with conditioning stimuli (+ C.S, purple). The GC-D related cue, CS_2 (10 ppm; used as a procedure control) and GG-related cues, 2PT (1:500), SBT (1:500) and pure mountain lion (Mt.Lion) urine are used as + C.S. In Phase 2, *ob* mice select between de.food and a novel unfamiliar food (new.food, in red). **c** Quantification of food preference ratio. Values obtained from mice are represented as mean ± SEM with aligned dot plots. Statistical significances of preference ratio are performed with *Z* tests, # $p < 0.05$, in yellow for de.food preference; non-significant if not mentioned. 14 to 18 animals were used per condition. The following pairs of spices were used: cinnamon (1%) *vs.* cocoa (2%); anise (1%) *vs.* oregano (2.4%).

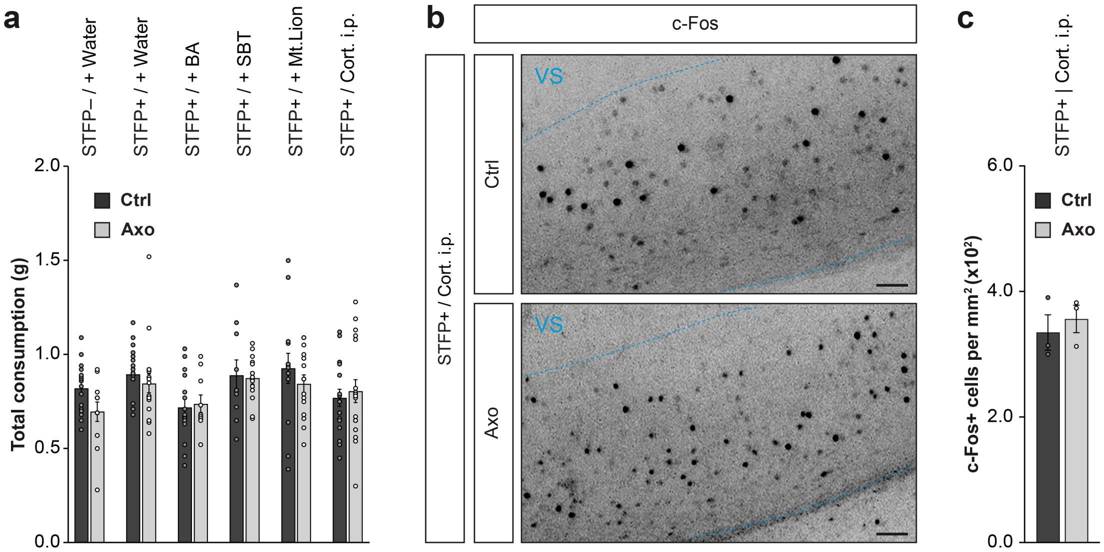

Supplementary Fig. 7 Impairment of an acquired food preference by chemical danger cues is not induced by a modification of the total food consumption or by a cortico-dependent VS activity. **a** The total food consumption of mice without or with STFP procedure and under the indicated conditioning association (STFP– or + / + C.S) or under intraperitoneal injection of 5.0 mg kg^{-1} corticosterone (Cort. i.p.) are shown. 10 to 18 animals were used per condition. The following pairs of spices were used: cinnamon 1% *vs.* cocoa 2%; anise 1% *vs.* oregano 2.4%; thyme 2% *vs.* basil 1.4%. **b** Representative c-Fos activity observed in the VS area (dashed blue lines) under STFP+ / Cort. i.p. condition. **c** Quantification of the density of c-Fos+ cells observed in VS under STFP+ / Cort. i.p. condition. 3 animals were used per Ctrl and Axo mice. (a and c) Values obtained from Ctrl (black) and Axo (grey) mice are represented as mean ± SEM with aligned dot plots.

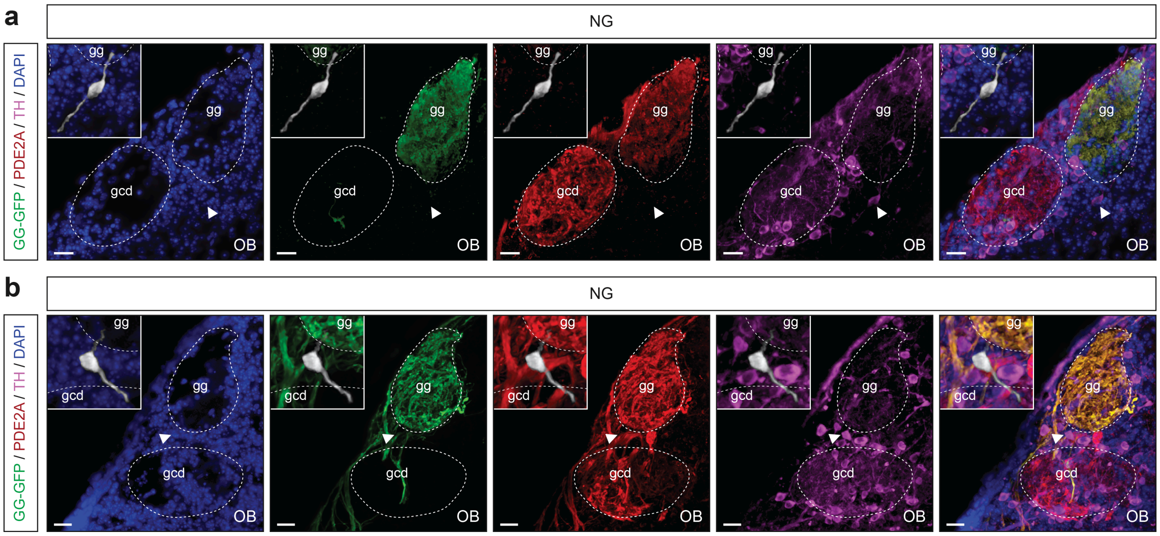

Supplementary Fig. 8 GG and GC-D circuitry are surrounded by periglomerular cells in the NG. (**a-b**) Representative immunostainings obtained from GCG–Cre–GFP mice (anti-GFP, GG-GFP in green; anti-PDE2A, PDE2A, in red). GG glomeruli (gg: GFP+ / PDE2A+) from the NG complex coexpress (in yellow) GFP and PDE2A. GC-D glomeruli (gcd: GFP- / PDE2A+) only express PDE2A. Periglomerular cells are observed in the NG complex thanks to their expression of the tyrosine hydroxylase (anti-TH, TH, in pink). White arrowheads indicate the localization of insets: zoom of a potential short axon cell (a) thanks to its distal localization and a potential interconnecting gg-gcd periglomerular cell (b), artificially colored in white. Scale bars are, 20 μm (a-b). Olfactory bulb: OB; glomeruli are delimited with dashed white lines. DAPI counterstains are shown in blue.
