# Supplementary material for: The Grueneberg ganglion controls odor-driven food choices in mice under threat
Source: Commun Biol. 2020 Sep 24;3:533. doi: 10.1038/s42003-020-01257-w (PMC7518244; doi:10.1038/s42003-020-01257-w)
Supplement: Supplementary file 2 — Description of Additional Supplementary Files [file 42003_2020_1257_MOESM2_ESM.pdf]

Description of Additional Supplementary Files

File Name: Supplementary Data 1
Description: The source data used to generate all graphs presented in the main figures of the result section of our manuscript (Figures 1-5).
