# Supplementary material for: The Grueneberg ganglion controls odor-driven food choices in mice under threat
Source: Commun Biol. 2020 Sep 24;3:533. doi: 10.1038/s42003-020-01257-w (PMC7518244; doi:10.1038/s42003-020-01257-w)
Supplement: Supplementary file 5 — Reporting Summary [file 42003_2020_1257_MOESM5_ESM.pdf]

nature research

Corresponding author(s): Marie-Christine Broillet

Last updated by author(s): Aug 18, 2020

Reporting Summary

Nature Research wishes to improve the reproducibility of the work that we publish. This form provides structure for consistency and transparency in reporting. For further information on Nature Research policies, see Authors & Referees and the Editorial Policy Checklist .

Statistics

For all statistical analyses, confirm that the following items are present in the figure legend, table legend, main text, or Methods section.

n/a	Confirmed	
☐	☒	The exact sample size (*n*) for each experimental group/condition, given as a discrete number and unit of measurement
☐	☒	A statement on whether measurements were taken from distinct samples or whether the same sample was measured repeatedly
☐	☒	The statistical test(s) used AND whether they are one- or two-sided<br>*Only common tests should be described solely by name; describe more complex techniques in the Methods section.*
☒	☐	A description of all covariates tested
☐	☒	A description of any assumptions or corrections, such as tests of normality and adjustment for multiple comparisons
☐	☒	A full description of the statistical parameters including central tendency (e.g. means) or other basic estimates (e.g. regression coefficient) AND variation (e.g. standard deviation) or associated estimates of uncertainty (e.g. confidence intervals)
☐	☒	For null hypothesis testing, the test statistic (e.g. *F*, *t*, *r*) with confidence intervals, effect sizes, degrees of freedom and *P* value noted<br>*Give P values as exact values whenever suitable.*
☒	☐	For Bayesian analysis, information on the choice of priors and Markov chain Monte Carlo settings
☒	☐	For hierarchical and complex designs, identification of the appropriate level for tests and full reporting of outcomes
☒	☐	Estimates of effect sizes (e.g. Cohen's *d*, Pearson's *r*), indicating how they were calculated

Our web collection on statistics for biologists contains articles on many of the points above.

Software and code

Policy information about availability of computer code

Data collection	Quantification of the food preference ratios were performed by scale measurements and collected in Microsoft Excel v14.7.7; For calcium imaging, visualizations and acquisitions of Fura2-loaded neurons were performed with Visitron MetaFluor v7.7.3.0; Measurements of corticosterone levels were done with the BioTek absorbance Reader ELx808 software; Acquisitions of confocal SP5 were done by using the Leica LAS v.AF software; Led-microscopy with AMG EVOS fl built-in software v1.4 were used for c-Fos pictures; Snapshots of representative behavioral assays were obtained with an infrared camera and its affiliated software, Sony Picture Motion Browser.
Data analysis	For calcium imaging experiments Visitron MetaFluor v7.7.3.0 off-line and Excel v14.7.7 were used to quantified calcium transients. Confocal maximum projections were treated with Bitplane Imaris v7.2.1; c-Fos images and their surface definition were done accordingly with NIH Fiji/ImageJ v1.48; Statistical analysis were performed with Microsoft Excel v14.7.7, the open source R v3.1.2 and GraphPad Prism 8.2.0.

For manuscripts utilizing custom algorithms or software that are central to the research but not yet described in published literature, software must be made available to editors/reviewers. We strongly encourage code deposition in a community repository (e.g. GitHub). See the Nature Research guidelines for submitting code & software for further information.

Data

Policy information about availability of data

All manuscripts must include a data availability statement. This statement should provide the following information, where applicable:

- Accession codes, unique identifiers, or web links for publicly available datasets
- A list of figures that have associated raw data
- A description of any restrictions on data availability

All data generated or analysed during this study are included in this published article (and its supplementary information files).

Field-specific reporting

Please select the one below that is the best fit for your research. If you are not sure, read the appropriate sections before making your selection.

- [x] Life sciences
- [ ] Behavioural & social sciences
- [ ] Ecological, evolutionary & environmental sciences

For a reference copy of the document with all sections, see nature.com/documents/nr-reporting-summary-flat.pdf

Life sciences study design

All studies must disclose on these points even when the disclosure is negative.

Sample size	For calcium imaging and histological experiments, the observed variability between individual recordings was modest and a minimum standard of a triplicate was thus necessary. For behavioral assays, sample size was chosen on the basis of pilot experiments and according to previously reported publications done in the field.
Data exclusions	According to our material and method description, only values obtained for a consumption ≥ 0.2 g were processed.
Replication	All findings were obtained from multiple and independent experiments. They were reliably reproduced.
Randomization	Equivalent sex-ratio were used for behavioral experiments. Female and male mice were selected randomly.
Blinding	For all behavioral assays the affiliated experimenter was aware of the conditions being tested as the tested olfactory cues were easily recognizable. Nevertheless, they were conducted blinded to phenotype (Ctrl vs. Axo) as the axotomization efficiency was only assessed at the end of the behavioral assays. Quantifications were performed using semi-automated methodology developed prior to data acquisition.

Reporting for specific materials, systems and methods

We require information from authors about some types of materials, experimental systems and methods used in many studies. Here, indicate whether each material, system or method listed is relevant to your study. If you are not sure if a list item applies to your research, read the appropriate section before selecting a response.

Materials & experimental systems

n/a	Involved in the study	
[ ]	[x]	Antibodies
[x]	[ ]	Eukaryotic cell lines
[x]	[ ]	Palaeontology
[ ]	[x]	Animals and other organisms
[x]	[ ]	Human research participants
[x]	[ ]	Clinical data

Methods

n/a	Involved in the study	
[x]	[ ]	ChIP-seq
[x]	[ ]	Flow cytometry
[x]	[ ]	MRI-based neuroimaging

Antibodies

Antibodies used

1st Antibodies:
*Goat anti-OMP; Wako; 1:1000; #544-10001, lot IUP1001, Antiserum. This antibody is widely used and has been validated by multiple previous publications that are listed on the manufacturer's website.

*Chicken anti-GFP; abcam; 1:600; #13970, lot GR3190550-6, Polyclonal. This antibody is commonly used and has been validated by multiple previous publications that are listed on the manufacturer's website.

*Rabbit anti-PDE2A; FabGennix; 1:400; #PD2A-101AP, lot FGI.159.Pld.Ig, Polyclonal. This antibody is commonly used and has been validated by multiple previous publications that are listed on the manufacturer's website.

*Mouse anti-TH; Immunostar; 1:2000; #22941, lot 1814001, Monoclonal. This antibody is widely used and has been validated by multiple previous publications that are listed on the manufacturer's website.

*Rabbit anti-c-Fos; Santa Cruz Biotechnology; 1:1000; sc-52, lot K0615, Polyclonal. This antibody is widely used and has been validated by multiple previous publications that are listed on the manufacturer's website.

*Mouse anti-c-Fos; Santa Cruz Biotechnology; 1:250; sc-166940, lot D2919, Monoclonal. This antibody is widely used and has been validated by multiple previous publications that are listed on the manufacturer's website.

2nd Antibodies:

*FITC-conjugated, Rabbit anti-Goat; Jackson ImmunoResearch; 1:200; #305-095-003, Polyclonal. This antibody has been validated in several previous publications that are listed on the manufacturer's website.

*FITC-conjugated, Donkey anti-Goat; Jackson ImmunoResearch; 1:200; #705-095-147, Polyclonal. This antibody has been validated in several previous publications that are listed on the manufacturer's website.

*Alexa Fluor 488-conjugated, Goat anti-Chicken; ThermoFisher; 1:200; #A11039, Polyclonal. This antibody has been validated in several previous publications that are listed on the manufacturer's website.

*Cy3-conjugated, Goat anti-Rabbit; Jackson ImmunoResearch; 1:200; #111-165-144, Polyclonal. This antibody has been validated in several previous publications that are listed on the manufacturer's website.

*Cy3-conjugated, Donkey anti-Rabbit; Jackson ImmunoResearch; 1:200; #711-165-152, Polyclonal. This antibody has been validated in several previous publications that are listed on the manufacturer's website.

*Alexa Fluor Plus 647-conjugated, Goat anti-Mouse; ThermoFisher; 1:200; #A32728, Polyclonal. This antibody has been validated in several previous publications that are listed on the manufacturer's website.

Validation

All antibodies are commercial and have been validated. Informations are available on the manufacturers' publicly accessible datasheets.

Animals and other organisms

Policy information about studies involving animals; ARRIVE guidelines recommended for reporting animal research

Laboratory animals

Adult (PM 1-6) and pups (PD 3-7) male and female C57BL/6 (Mus musculus), GCG–Cre–GFP (lines 52 and 43) and OMP-GFP mice were used. In transgenic GCG–Cre–GFP mice, the expression of the green fluorescent protein (GFP) is driven by the GC-G gene which allows the selectively expression of this marker in GG neurons and axons. In the gene-targeted mouse strain OMP-GFP , the GFP is used as a histological reporter of mature olfactory sensory neurons expressed under the control of the olfactory marker protein (OMP) promoter.

Wild animals

The study did not involve wild animals.

Field-collected samples

The study did not involve samples collected from the field.

Ethics oversight

The animal experimental procedures were in accordance with the Swiss legislation and approved by the EXPANIM committee of the Lemanique Animal Facility Network and the veterinary authority of the Canton de Vaud (SCAV).

Note that full information on the approval of the study protocol must also be provided in the manuscript.
